# Clinical value of AEF in the post-processing technique of liver perfusion-like phase III enhanced CT scan in evaluating the degree of liver function impairment in patients with Hepatitis-B cirrhosis

**DOI:** 10.12669/pjms.38.7.5362

**Published:** 2022

**Authors:** Nannan Wang, Zhilei Sun

**Affiliations:** 1 Nannan Wang Department of Radiology, The Affiliated Huai’an Hospital of Xuzhou Medical University, Huai’an, 223002, Jiangsu, China; 2 Zhilei Sun Department of Radiology, The First Affiliated Hospital of Soochow University, Suzhou, 215000, Jiangsu, China

**Keywords:** Liver phase III enhanced CT scan, Post-processing technique, CT arterial enhancement fraction, Hepatitis B cirrhosis, Degree of liver function impairment

## Abstract

**Objectives:**

To investigate the value of CT arterial enhancement fraction (AEF) in the post-processing technique of liver perfusion-like phase III enhanced CT scan in evaluating the degree of liver function impairment in patients with hepatitis B cirrhosis.

**Methods:**

The study included 85 patients with hepatitis B cirrhosis admitted to the Department of Radiology, The Affiliated Huai’an Hospital of Xuzhou Medical University from May 2018 to October 2020 were selected as the experimental group, and 71 patients with liver fibrosis during the same period were selected as the control group. All patients underwent routine liver CT phase III perfusion scan, and hepatic AEF (hAEF) and liver/spleen ratio (H/S) were compared between the two groups to analyze the differential value of hAEF and H/S for liver fibrosis and hepatitis B cirrhosis. Patients were divided into the mild group (Grade-A) and the severe group (Grade-B and C) according to Child-Pugh grading. hAEF and H/S values of the two groups were compared, and the evaluation value of AEF on the degree of impairment of hepatitis B cirrhosis was analyzed.

**Results:**

hAEF and H/S values of the experimental group were greater than those of the control group (*P*<0.05), and the AUCs of hAEF and H/S values for distinguishing hepatitis B and cirrhosis were 0.727 (95%CI: 0.650-0.795) and 0.791 (95%CI: 0.718-0.852), respectively. Moreover, hAEF and H/S values of the severe group were greater than those of the mild group (*P*<0.05), and the AUCs of hAEF and H/S values in evaluating the degree of liver function impairment were 0.746 (95%CI: 0.627-0.834) and 0.770 (95%CI: 0.705-0.928), respectively.

**Conclusions:**

AEF boasts the value of differentiating liver fibrosis and cirrhosis, and of evaluating the degree of liver function impairment in patients with hepatitis B cirrhosis.

## INTRODUCTION

Cirrhosis is a chronic end-stage liver disease with hepatitis B as one of its primary causes. Patients with such a disease are often accompanied by diffuse hepatic fibrosis, pseudolobules and regenerated nodules. In the wake of the progression of the disease, patients may develop complications such as ascites and gastrointestinal bleeding, which seriously affect the life and health of the patients.^1^ It has been pointed out in some studies that the adoption of appropriate methods to evaluate patients’ liver function is beneficial to early intervention by physicians, thus improving the prognosis.^2^ With the development of imaging technology, CT perfusion examination has been extensively applied in the examination of liver diseases by virtue of its accurate reflection of blood perfusion in the progress of cirrhosis and the judgment of the degree of liver function impairment.^3^ The arterial enhancement fraction (AEF) , which is defined as the ratio of hepatic arterial perfusion to the total hepatic perfusion, can provide noninvasive assessment of hepatic perfusion. Previous studies have shown that, hepatic AEF was closely associated with the severity and prognosis of patients with liver cirrhosis, which have the potential to estimate the liver function in liver cirrhosis patients quantitatively and effectively.^4^ However, its value in judging the degree of liver function impairment in patients with hepatitis B cirrhosis is still controversial. Therefore, in this study, CT AEF in the post-processing technique of liver perfusion-like phase III enhanced CT scan in patients with different liver diseases and varying degrees of liver function damage were analyzed, so as to investigate its value in evaluating the degree of liver function impairment in patients with hepatitis B cirrhosis.

## METHODS

A total of 85 patients with hepatitis B cirrhosis admitted to the Department of Radiology, The Affiliated Huai’an Hospital of Xuzhou Medical University from May 2018 to October 2020 were selected as the experimental group, and 71 patients with liver fibrosis during the same period were selected as the control group, all of which met the diagnostic criteria of hepatitis B in Guidelines for the Prevention and Treatment of Chronic Hepatitis B (Version 2015).^5^ The sample size required for each group was calculated by the formula:


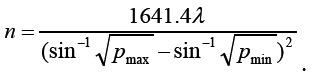


### Ethical Approval

The study was approved by the Institutional Ethics Committee of the Affiliated Huai’an Hospital of Xuzhou Medical University on August 2020 (No. [2020]35), and written informed consent was obtained from all participants.

### Inclusion criteria:

- Patients with confirmed diagnosis;
- Patients with complete upper abdominal plan CT scan and phase III enhanced examination data.

### Exclusion criteria:

- Patients with malignant tumor diseases;
- Patients with other types of liver diseases such as hepatitis A and fatty liver;
- Patients with acute liver failure;
- Patients with cardiac and renal insufficiency;
- Patients with contraindications to the use of contrast agents;
- Patients with contraindications to CT examination;
- Patients with a history of abdominal surgery. Patients in the experimental group were diagnosed with cirrhosis by imaging examination.

### CT examination methods

Patients were fasted for 4-6hour before the examination and drank 500ml water 15 minutes before the examination to replenish the gastrointestinal tract. The second-generation dual-source CT scanner of Siemens was used for CT scanning, and 20G intravenous cannula indwelling needle was reserved before examination. Patients were placed in supine position with hands raised to the sides of the headrest. Chest breathing was instructed during the CT scan, while the abdomen was pressurized and immobilized with a wide abdominal band, with a scan range from the top of the diaphragm to the lower margin of the liver. Ioprolamide contrast agent (Bayer Healthcare LLC, Batch No. H20080534) was injected at the median cubital vein with a dose of 1.5 mL/kg (calculated by body mass) and a flow rate of 4.0ml/s. Spiral scanning was used for scanning, and the scanning was performed at the arterial, portal and delay stages 28 Seconds, 60 Second and 180 Second after the injection of contrast agent. The tube voltage was 80-100kV, and the tube current was 160-250mAs, with a pitch of 0.8, a layer thickness of 5mm, a layer spacing of 5mm, a rotation time of 0.28s, and a matrix of 512*512.

### Image quality Requirements

Arterial phase images should conform to the significant enhancement of the abdominal aorta and hepatic artery, and there should be no significant enhancement or slight enhancement of the portal vein. Portal vein phase images should be consistent with the significant enhancement of the portal vein, with a degree of enhancement higher than that of the abdominal aorta; The respiratory movement in each phase of the scan image has little influence on the size, position and shape of the liver, and can be automatically registered.

### AEF value detection

Phase-III CT liver images were imported into CT Kinetics software for processing, and liver dual input model was selected to fit and calculate liver perfusion parameters (hepatic AEF (hAEF), liver/spleen ratio (H/S)). AEF=[(HU artery stage - HU plain scan CT)/(HU _venous stage_ - HU _plain scan_ CT)]*100%. The best layer displayed at the hepatic portal was taken as the middle layer, and the calculation layer was set to about 10 layers to ensure that most of the eight segments of the liver could be calculated. Three regions of interest (ROIs) were selected by the attending radiologist at each segment of the liver using a multi-point method, with an area of approximately 50-55 mm^2^ each. AEF-like perfusion parameters of 3 ROIs were measured for each liver segment and averaged. Large vessels and branching vessels visible to the naked eye should be avoided when selecting ROIs, so as to reduce the interference of partial volume effect and other factors. The average perfusion parameters of the whole liver can be obtained by adding the AEF values of each segment and dividing by the number of segments.

### Observation Indicators

hAEF and H/S values of the experimental group and the control group were compared to analyze its differential value for liver fibrosis and hepatitis B cirrhosis. Patients were divided into the mild group (Grade-A) and the severe group (Grade-B and grade C) according to Child-Pugh grading. hAEF and H/S values of the two groups were compared, and the evaluation value of AEF on the degree of impairment of hepatitis B cirrhosis was analyzed.

### Statistical Analysis

All the data were processed by SPSS17 software. Measurement data were expressed as %, and χ2 test was used to compare the difference between groups. Measurement data were expressed as (*x̅*±*s*), and two independent sample t-test was adopted for comparison of differences between the two groups. ROC curve was used to analyze the differential value of hAEF and H/S values in hepatitis B cirrhosis and evaluate the degree of impairment in hepatitis B cirrhosis. *P*<0.05 indicates a statistically significant difference.

## RESULTS

In the experimental group, there were 51 males and 34 females, aged from 41 to 69 years, with an average of (56.82±5.95) years; The course of hepatitis B was 2-8 years, with an average of (4.10±0.97) years. Child-pugh classification: 46 cases of Grade-A, 21 cases of Grade-B, and 18 cases of grade C. In the control group, there were 40 males and 31 females, aged 40-72 years old, with an average of (57.67±6.11) years; The course of hepatitis B was 1-7 years, with an average of (3.82±0.96) years. No significant difference was observed in gender, age and course of hepatitis B course between the two groups (*P*>0.05).hAEF and H/S values in the experimental group were higher than those in the control group (*P*<0.05), and hAEF and H/S values of the severe group were greater than those of the mild group (*P*<0.05), as shown in Table-I.

**Table I:** Comparison of hAEF and H/S values between the experimental and control groups, and between the mild group and the severe group (*x̅*±*s*).

Group	n	hAEF (%)	H/S	Group	n	hAEF(%)	H/S
Experimental group	85	46.22±6.24	0.85±0.09	Mild group	46	43.69±6.8	0.74±0.12
Control group	71	41.07±5.61	0.72±0.08	Severe group	39	49.20±7.1	0.98±0.15
t		5.373	9.446	t		3.648	8.194
P		<0.001	<0.001	P		<0.001	<0.001

**Fig.1:**
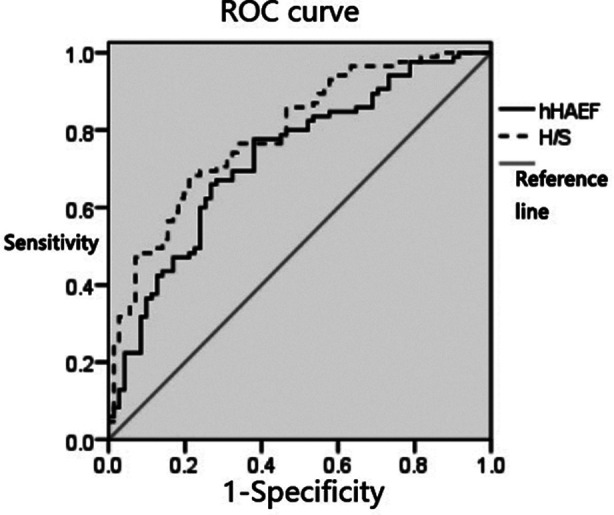
ROC curve of hAEF and H/S values in differentiating hepatitis B and liver cirrhosis

**Fig.2:**
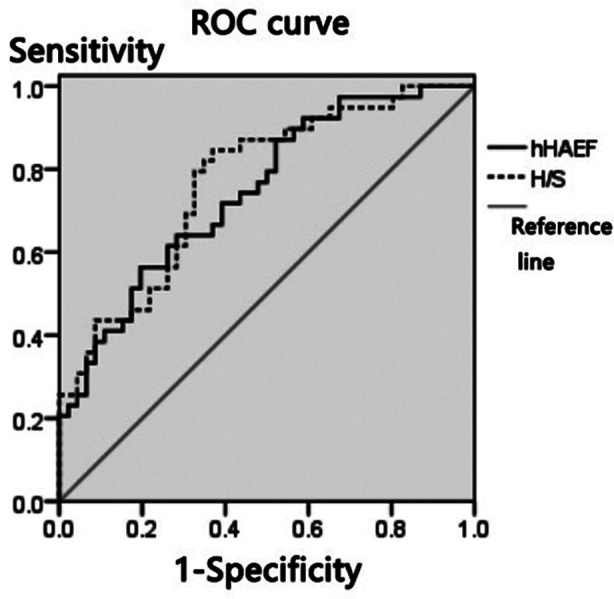
ROC curve of hAEF and H/S values to evaluate the degree of liver function impairment in patients.

The AUCs of hAEF and H/S values for differentiating hepatitis B and liver cirrhosis were 0.727 and 0.791, respectively, as shown in Table-II and Fig.1.The AUCs of hAEF and H/S values to evaluate the degree of liver function impairment in patients were 0.716 and 0.770, respectively, as shown in Table-III and Fig.2.

**Table II:** Differential value of hAEF and H/S values for hepatitis B and liver cirrhosis.

Indicators	Cut-off value	AUC	SE	95%CI
hAEF	42.55%	0.727	0.041	0.650~0.795
H/S	0.81	0.791	0.036	0.718~0.852

**Table III:** Evaluation value of hAEF and H/S values for the degree of liver function impairment in patients

Indicators	Cut-off value	AUC	SE	95%CI
hAEF	46.95%	0.746	0.053	0.627~0.834
H/S	0.87	0.770	0.051	0.705~0.928

## DISCUSSION

Hepatitis B is one of the commonly seen chronic liver diseases with a high incidence in China. It is estimated that nearly 10% of the population in China is positive for hepatitis B virus surface antigen, and approximately 3% of patients will progress to liver cirrhosis.^6^,^7^ Patients with early liver cirrhosis have liver fibrosis that is reversible, but those with advanced liver cirrhosis have worsening liver fibrosis that is irreversible, often with a poor prognosis.^8^,^9^ Therefore, it is crucial to explore early diagnosis methods for liver fibrosis and cirrhosis to improve the prognosis. Studies have pointed out that abnormal liver blood perfusion is the primary pathophysiological change of cirrhosis^10^, suggesting that patients’ condition may be judged by detecting liver blood perfusion. AEF, which refers to the ratio of the absolute increase in the arterial phase to the absolute increase in the portal phase, can indirectly reflect the blood supply of the hepatic artery. It was found in this study that that hAEF and H/S values of the experimental group were greater than those of the control group, indicating that patients with liver cirrhosis have abnormal liver blood perfusion, which can be attributed to the fact that liver cirrhosis may cause damage of normal lobular structure in liver parenchyma, aggravation of liver fibrosis and nodular regeneration, thus increasing intrahepatic vascular resistance and affecting hepatic blood perfusion.^11^,^12^ In this study, hAEF and H/S values were found to be of differential value for liver fibrosis and cirrhosis, indicating that patients’ condition could be judged by detecting hepatic blood perfusion.

CT perfusion imaging reflects the degree of liver function impairment by quantifying changes in hepatic circulation hemodynamics. Patients with liver cirrhosis often suffer from severe liver fibrosis, in which deposition of collagen tissue in the liver in the sinus space leads to increased intrahepatic blood flow resistance, blocked portal venous return, and increased hAEF.^13^-^15^ Patients with severe cirrhosis suffer from splenic venous return obstruction and even the emergence of hepatic blood flow due to the increase of portal vein pressure, while the blood stasis and intradermal blood stasis at portal vein stage will result in decreased splenic blood flow and increased H/S.^16^-^18^ It was found by this study that the hAEF and H/S values of the severe group were greater than those of the mild group, suggesting that the hAEF and H/S values may be related to the degree of liver function impairment in patients. To explain this, mild liver function impairment mainly manifests as part of hepatocyte degeneration, necrosis, and mild proliferation of fibrous tissue, but there is no connection between the proliferating fibrous tissues. As the disease worsens, patients with moderate to severe cirrhosis tend to have obvious proliferation of collagen fibers and interconnection of the fibrous separation between the central lobules and the portal area. Regenerated nodules of the liver can compress the peripheral hepatic vein and portal vein branches, resulting in vascular stenosis or even occlusion, and increased vascular resistance. In this way, abnormal blood perfusion of portal vein and portal artery occurs.^19^-^21^ It has been pointed out by some studies that significant differences were observed between hAEF of cirrhosis of different severity and that of normal control group^22^,^23^, suggesting that the severity of patients’ condition may be determined by testing hAEF. In this study, hAEF and H/S values of patients with different Child-Pugh grades were analyzed, and the results showed that the AUC of hAEF and H/S values in evaluating the degree of liver function impairment of patients were 0.716 and 0.770, respectively, indicating that hAEF and H/S values are valuable in evaluating the degree of liver function impairment of patients with hepatitis B cirrhosis. It can be concluded that the degree of liver function impairment can be evaluated by detecting hAEF and H/S values of patients, and thus early targeted intervention can be given to patients.

### Limitations of this study

A small sample was included and the proportion of patients with different levels of liver function impairment varied, which may lead to deviations in the results. In response to this, more samples will be included in the follow-up study to further investigate the relationship between AEF in the post-processing technique of liver perfusion-like phase III enhanced CT scan and liver function impairment in patients with hepatitis B cirrhosis.

## CONCLUSION

AEF is valuable in differentiating liver fibrosis and cirrhosis, and of evaluating the degree of liver function impairment in patients with hepatitis B cirrhosis.

### Authors’ Contributions:

**NW:** Designed this study, prepared this manuscript, are responsible and accountable for the accuracy and integrity of the work.

**ZS:** Collected and analyzed clinical data.
